# Anti-parasite therapy drives changes in human visceral leishmaniasis-associated inflammatory balance

**DOI:** 10.1038/s41598-017-04595-8

**Published:** 2017-06-28

**Authors:** Théo Araújo-Santos, Bruno B. Andrade, Leonardo Gil-Santana, Nívea F. Luz, Priscila L. dos Santos, Fabrícia A. de Oliveira, Meirielly Lima Almeida, Roseane Nunes de Santana Campos, Patrícia T. Bozza, Roque P. Almeida, Valeria M. Borges

**Affiliations:** 1 Instituto Gonçalo Moniz, Fundação Oswaldo Cruz, 40296-710 Salvador, Brazil; 2 Centro das Ciências Biológicas e da Saúde, Universidade Federal do Oeste da Bahia, 47808-021 Barreiras, Brazil; 3 Multinational Organization Network Sponsoring Translational and Epidemiological Research (MONSTER) Initiative, Fundação José Silveira, 40210-320 Salvador, Bahia Brazil; 4 Curso de Medicina, Faculdade de Tecnologia e Ciências, 40290-150 Salvador, Brazil; 5 Universidade Salvador (UNIFACS), Laureate Universities, 41720-200 Salvador, Brazil; 6 Escola Bahiana de Medicina e Saúde Pública, 40290-000 Salvador, Brazil; 7 Departamento de Medicina e Patologia, Hospital Universitário, Universidade Federal de Sergipe, 49060-100 Aracaju, Sergipe Brazil; 8 Instituto Oswaldo Cruz, Fundação Oswaldo Cruz, 21045-900 Rio de Janeiro, Rio de Janeiro Brazil; 9 Instituto Nacional de Ciência e Tecnologia de Investigação em Imunologia, São Paulo, Brazil; 10 Faculdade de Medicina da Bahia, Universidade Federal da Bahia, 40055-150 Salvador, Bahia Brazil

## Abstract

Visceral leishmaniasis (VL) remains a major public health problem worldwide. Cytokine balance is thought to play a critical role in the development of this disease. Here, we perform a prospective exploratory study addressing whether simultaneous assessment of circulating levels of different lipid mediators and cytokines could highlight specific pathways involved with VL pathogenesis. VL patients displayed substantial increases in serum levels of Prostaglandin F_2_α (PGF_2_α), Leukotriene B_4_ (LTB_4_), Resolvin D1 (RvD1), IL-1β, IL-6, IL-8, IL-10, IL-12p70 and TNF-α compared with uninfected endemic control group, while exhibiting decreased levels of TGF-β1. Hierarchical cluster analysis of the prospective changes in the expression level of theses parameters upon anti-*Leishmania* treatment initiation revealed that the inflammatory profile observed in active disease gradually changed over time and was generally reversed at day 30 of therapy. Furthermore, not only the individual concentrations of most of the inflammatory biomarkers changed upon treatment, but the correlations between those and several biochemical parameters used to characterize VL disease activity were also modified over time. These results demonstrate that an inflammatory imbalance hallmarks active VL disease and open perspective for manipulation of these pathways in future studies examining a potential host-directed therapy against VL.

## Introduction

Visceral leishmaniasis (VL) is a chronic infection caused by some species of *Leishmania* parasites that has been associated with high morbidity and mortality in many developing countries. The most prevalent symptoms of VL are fever, substantial weight loss, splenomegaly and hepatomegaly. If left untreated, up to 100% of all VL patients can die within two years^1^. Possible mechanisms linked to increased disease severity in VL are still unknown, but unfettered inflammation is thought be a key determinant^2, 3^. An immunological hallmark of VL is the absence of effective Th1 immune responses against *Leishmania* parasites, with high circulating levels of IL-10^4–6^. Understanding the immune determinants of VL is key in driving development of innovative host-directed therapies to optimize leishmanicidal treatment efficacy^7, 8^.

Lipid mediators have been described to contribute to the inflammatory environment in a number of diseases, including leishmaniasis^9, 10^. Recently, our group has described unique expression profiles of the lipid mediator pathways in patients with localized or mucosal cutaneous leishmaniasis^11^. PGE_2_ levels were shown to be increased whereas PGF_2_α levels were decreased in localized cutaneous leishmaniasis compared with healthy endemic controls^11^. Further investigations revealed that plasma concentrations of the lipid mediator resolvin D1 are substantially increased in patients with diffuse cutaneous leishmaniasis compared to that in individuals with localized disease^12^. Another study from our group has demonstrated that PGF_2_α drives immune evasion of *Leishmania infantum* during *in vitro* infection of macrophages^13^. More recently, we have described that dogs with severe canine VL display decreased circulating levels of LTB_4_ and PGE_2_ compared to those with mild or asymptomatic disease^14^, reinforcing the idea that lipid mediators are tightly associated with VL disease progression. Whether these host-derived lipid mediators play an important role in the pathogenesis of human VL is unknown.

In the present study, we perform an exploratory prospective investigation examining the expression profile of markers of inflammation, immune activation as well as lipid mediators in serum from patients with VL. We first compared serum concentrations of biomarkers between individuals with active disease (treatment naïve) and healthy endemic controls. Our findings revealed a distinct biosignature of active VL hallmarked by increased serum levels of PGF_2_α, LTB_4_, RvD1, TNF-α, IL-1β, IL-6 and IL-8, IL-10 and IL-12p70, while exhibiting lower concentrations of TGF-β1. Moreover, we found that the inflammatory profile of VL was mostly reversed post initiation of leishmanicidal treatment. These findings demonstrate that an inflammatory imbalance characterizes active VL disease and identify potential targets for host-directed therapies.

## Methods

### Ethics Statement

This study was approved by Institutional Review Board of the Federal University of Sergipe, Brazil (license number: 04587312.2.0000.0058). All clinical investigations were conducted according to the Declaration of Helsinki. Written informed consent was obtained from all participants or legal guardians.

### Study design

Serum samples were obtained from patients with confirmed VL diagnosis (n = 50) admitted at the Infectious Diseases Reference Hospital of the Federal University of Sergipe. The diagnostic criteria used for inclusion in the study were identification of *Leishmania* in bone marrow aspirates by direct microscopic exam and/or culture in NNN media (Sigma-Aldrich, St Louis, MO) as well as positive rK39 serology test (Kalazar Detect® Rapid Test; InBios International Inc., Seattle, WA). In addition, all patients enrolled were treatment naïve and had no history of previous VL diagnosis. After diagnosis, patients received standard Antimony treatment (20 mg Sb^v^/Kg/day) intravenously. In our clinical study, patients received treatment for 20 days following the Brazilian guidelines for management of VL and samples were obtained after 15 days of treatment initiation (during the anti-parasite therapy) and after 30 days of treatment initiation (10 days after treatment completion). Additional blood samples were collected to perform white blood cell counts, and quantification of hematological and biochemical parameters (indicated in the text results) at the clinical laboratory of the hospital from which patients were recruited. Serum samples were also collected from a group of individuals (either household contacts or relatives) living in the same area as the patients but with no signs of clinical disease (endemic healthy controls; n = 16). Serum samples were collected and stored at −80 °C until the use in immunoassays.

### Immunoassays

Serum levels of IL-1β, IL-6, IL-8, IL-12p70 and TNFα were measured using the commercially available Human Inflammation set, Cytometric Bead Array (BD Biosciences Pharmingen, San Diego, CA) according to the manufacturer’s protocol. The flow cytometric assay was performed and analyzed by a single operator, and standard curves were derived from cytokine standards. PGE_2_, PGF_2α_, LTB_4_ and RvD1 levels were quantified by enzyme-linked immunoassay, according to the manufacturer’s instructions (Cayman Chemical, Ann Arbor, MI). After acidification to activate latent TGF-β1 followed by neutralization, total TGF-β1 was measured in the serum using ELISA (R&D systems, Minneapolis, MN), according to the manufacturer’s instructions.

### Statistical analysis

Median values with interquartile ranges (IQR) were used as measures of central tendency. In unmatched analyses, the Mann-Whitney test (for two groups) or Kruskal-Wallis with Dunn’s multiple comparisons ad hoc test (for more than two groups) were used to compare continuous variables. The Fisher’s (two groups) or chi-square (more than two groups) tests were used to compare variables displayed as percentage. The Wilcoxon matched-pairs test was performed to estimate statistical significance before and at different time points after leishmanicidal treatment initiation. Unsupervised two-way hierarchical cluster analysis (Ward’s method) with 100X bootstrap were utilized to test whether VL patients at different timepoints of leishmanicidal treatment and endemic healthy controls could be grouped separately based on the overall expression profile of serum markers. Spearman correlations matrices were built to compare the association profile between serum markers and several biochemical parameters. P-values were adjusted for multiple measurements/comparisons using Bonferroni’s method. The statistical analyses were performed using GraphPad Prism 6.0 (GraphPad Software Inc., USA) and JMP 11.0 (SAS, Cary, NC, USA) software. A p-value < 0.05 was considered statistically significant.

## Results

### Baseline characteristics

At the time of enrollment, the majority of the study participants were children and from male gender (Table 1). In addition, all study participants screened negative for HIV infection. Both groups of VL patients and healthy controls were similar with regard to age (p = 0.096; Table 1). Frequency of male individuals was higher in the group of VL patients compared with healthy controls (56% vs. 25% respectively, p = 0.0443; Table 1). As expected, at the study baseline, treatment-naïve VL patients presented with intense anemia and thrombocytopenia (Table 1). In addition, the VL patients presented a significant leucopenia with neutropenia (Table 1).

**Table 1:** Baseline characteristics of the study participants.

Characteristic	Unit	Healthy control	Visceral Leishmaniasis	P-value
N		16	50	
Male	no. (%)	4 (25)	28 (56)	0.044
Age	years	16.5 (9.5–21)	8.5 (3.7–17.5)	0.096
Hb	g/dL	12.9 (12.1–13.8)	8.6 (7.5–9.7)	<0.0001
RBC	10^3^/L	4.7 (4.4–5.0)	3.5 (3.2–3.9)	<0.0001
Platelets	10^3^/L	265 (225–277)	166 (129–220)	<0.0001
WBC	10^9^/L	7.11 (6.73–8.62)	2.80 (1.90–3.80)	<0.0001
Neutrophils	10^9^/L	3.48 (2.98–3.84)	0.82 (0.57–1.31)	<0.0001
Monocytes	10^9^/L	0.68 (0.49–0.76)	0.38 (0.29–0.55)	0.0005
Lymphocytes	10^9^/L	2.89 (2.42–3.69)	1.45 (0.88–1.99)	<0.0001

Values represent median and interquartile ranges, except for gender distribution, which is shown as percentage. Data were analyzed using the Mann-Whitney *U* test (continuous variables) and the Fisher’s exact test (frequency analysis). Abbreviations: Hb, hemoglobin; RBC, red blood cell count; WBC, white blood cell count.

### Systemic inflammatory imbalance during active VL

Previous studies have described high circulating levels of proinflammatory cytokines in patients with VL^3^. In the present study, we simultaneously assessed serum levels of two major prostanoids already described by our group in tegumentary leishmaniasis, PGE_2_^11, 15^ and PGF_2_α^13^, as well as other lipid mediators such as LTB_4_ and RvD1^11^. In addition, we assessed levels of key cytokines associated with inflammation and also previously linked to VL pathogenesis IL-1β^2^, IL-6^2, 4, 16–18^, IL-8^2, 4, 19^, IL-10^4–6, 16, 20–26^, IL-12p70^4, 22^, TNF-α^4, 18, 21, 27–29^, and TGF-β1^19, 24, 30, 31^ (Table 2). We found that VL patients exhibited a very distinct expression profile compared with uninfected healthy controls (Table 2). We observed that TGF-β1 levels were significantly higher in healthy controls compared with individuals with VL (Table 2). On the converse, serum concentrations of IL-1β, IL-6, IL-8, IL-10, IL-12p70, PGF_2_α and TNF-α were substantially higher in VL patients than in those from the healthy control group (Table 2). Interestingly, contrasting with previous reports from our group which showed increased PGE_2_ levels in localized cutaneous leishmaniasis compared to uninfected controls^11^, we observed that serum concentrations of this prostanoid were undistinguishable between VL patients and controls (Table 2). We next tested if differences in the inflammatory profile described here could be explained based on the differential frequency of male participants between the study groups (VL and healthy controls). Notably, we found no statistically significant differences between male and female subjects (Supplemental Table 1), indicating that gender did not dramatically influence the results on inflammatory markers described here. These findings highlight a biosignature of inflammatory markers in treatment-naïve VL.

**Table 2:** Concentrations of serum markers in the study population.

Marker	Units	Healthy control	Visceral Leishmaniasis	P-value
TGF-β1	pg/mL	56.5 (48.4–67.9)	21.8 (17.4–38.9)	<0.0001
PGE_2_	pg/mL	0.7 (0.4–0.8)	0.7 (0.4–1.0)	0.669
PGF_2_α	ng/mL	0.4 (0.2–0.7)	5.3 (4.0–6.5)	<0.0001
LTB4	ng/mL	5.17 (3.6–9.3)	16.8 (12.6–29.7)	<0.0001
RvD1	ng/mL	82.5 (45.5–115)	200 (92.3–369)	<0.0001
TNF-α	pg/mL	1.0 (0.6–1.9)	21.7 (19.9–27.1)	<0.0001
IL-1β	pg/mL	0.8 (0.4–1.2)	17.0 (14.6–22.2)	<0.0001
IL-6	pg/mL	1.2 (0.6–2.1)	6.5 (4.3–9.4)	<0.0001
IL-8	pg/mL	7.0 (5.3–14.0)	23.8 (19.2–29.8)	<0.0001
IL-10	pg/mL	2.0 (1.5–3.2)	58.2 (37.7–78.5)	<0.0001
IL12p70	pg/mL	1.8 (1.5–2.3)	8.5 (7.4–9.8)	<0.0001

Values represent median and interquartile ranges. Data were analyzed using the Mann-Whitney *U* test (continuous variables).

### Changes in the inflammatory profile of VL patients upon antileishmanial treatment initiation

After study enrollment, all VL patients underwent pentavalent antimonial treatment following Brazilian national guidelines^1^ for 20 days and blood samples were collected at 15 day of therapy as well as 10 days after therapy completion (day 30 after therapy initiation). Relevant hematological and biochemical parameters such as platelet and neutrophil counts and levels of hemoglobin and albumin gradually increased following days of treatment, achieving the highest values after 30 days of treatment initiation (Table 3).

**Table 3:** Hematological and biochemical parameters from patients with visceral leishmaniasis before and after leishmanicidal treatment initiation.

Parameter	Unit	Pre-treatment	Day 15	Day 30	P-value	Post-test result
Hb	g/dL	8.7 (7.6–10.0)	9.8 (8.6–10.3)	10.6 (9.8–11.4)	<0.0001	*, #
RBC	10^3^/L	3.6 (3.2–4.1)	4.0 (3.4–4.3)	4.1 (3.6–4.3)	0.0005	*, #
Platelets	10^3^/L	167 (134–222)	201 (172–296)	254 (218–323)	<0.0001	*, #, $
WBC	10^9^/L	2.80 (1.90–3.70)	3.39 (2.71–4.68)	5.70 (4.51–7.32)	<0.0001	*, #, $
Neutrophils	10^9^/L	0.81 (0.58–1.18)	1.11 (0.77–1.57)	2.16 (1.55–3.02)	<0.0001	*, #
Monocytes	10^9^/L	0.37 (0.28–0.51)	0.44 (0.33–0.59)	0.48 (0.36–0.63)	0.7852	n.s.
Lymphocytes	10^9^/L	1.45 (0.85–2.02)	1.65 (1.22–2.39)	2.63 (1.6–3.43)	<0.0001	*, #, $
Albumin	g/dL	2.5 (2.2–3.0)	2.9 (2.4–3.5)	3.8 (3.2–4.1)	<0.0001	*, #, $
Globulin	g/dL	4.9 (3.8–5.9)	4.8 (4.3–6.3)	4.5 (3.8–5.4)	0.4081	n.s.
Total protein	g/dL	13.5 (12.4–14.9)	12.6 (12.1–13.8)	12.7 (12.0–13.5)	0.0652	n.s.
aPTT	g/dL	37.1 (33.5–42.6)	38.6 (35.7–42.4)	37.4 (34.2–39.6)	0.9800	n.s.
AST	U/L	58 (38–87)	60 (46–100)	44 (38.2–52.7)	0.8454	n.s.
ALT	U/L	36 (26.2–70.2)	61 (42.5–104)	46 (32.5–56)	0.1510	n.s.
ALP	U/L	153 (109–273)	196 (137–320)	211.5 (149–244.5)	0.4374	n.s.
GGT	U/L	63.5 (20–145.5)	103 (33.5–215)	53.5 (26–107.8)	0.0819	n.s.
Amilase	U/dL	45.5 (34.2–70.5)	84 (61–152)	70.5 (53–85.6)	0.2207	n.s.
Urea	mg/dL	22 (16–27.2)	22 (17–30)	22 (17–31)	0.3944	n.s.
Creatinine	mg/dL	0.5 (0.4–0.7)	0.5 (0.3–0.7)	0.5 (0.3–0.7)	0.9758	n.s.

Values represent median and interquartile ranges. Log_10_ transformed data (which presented Gaussian distribution) were analyzed using one-way ANOVA with Tukey’s multiple comparisons post-test. Column with P-values represent the one-way ANOVA comparisons. Column with P-values represent the one-way ANOVA comparisons. P-values from post-test are represented by the following: *p < 0.05 in Pre-treatment vs. Day 15; ^#^p < 0.05 in Pre-treatment vs. Day 30; ^$^p < 0.05 in Day 1`s. Day 30; n.s. nonsignificant. Abbreviations: aPPT, activated partial thromboplastin time; ALP, alkaline phosphatase; ALT, alanine aminotransferase; AST, aspartate aminotransferase; GGT, Gamma-glutamyl transpeptidase; Hb, hemoglobin; RBC, red blood cell count; WBC, white blood cell count.

**Figure 1:**
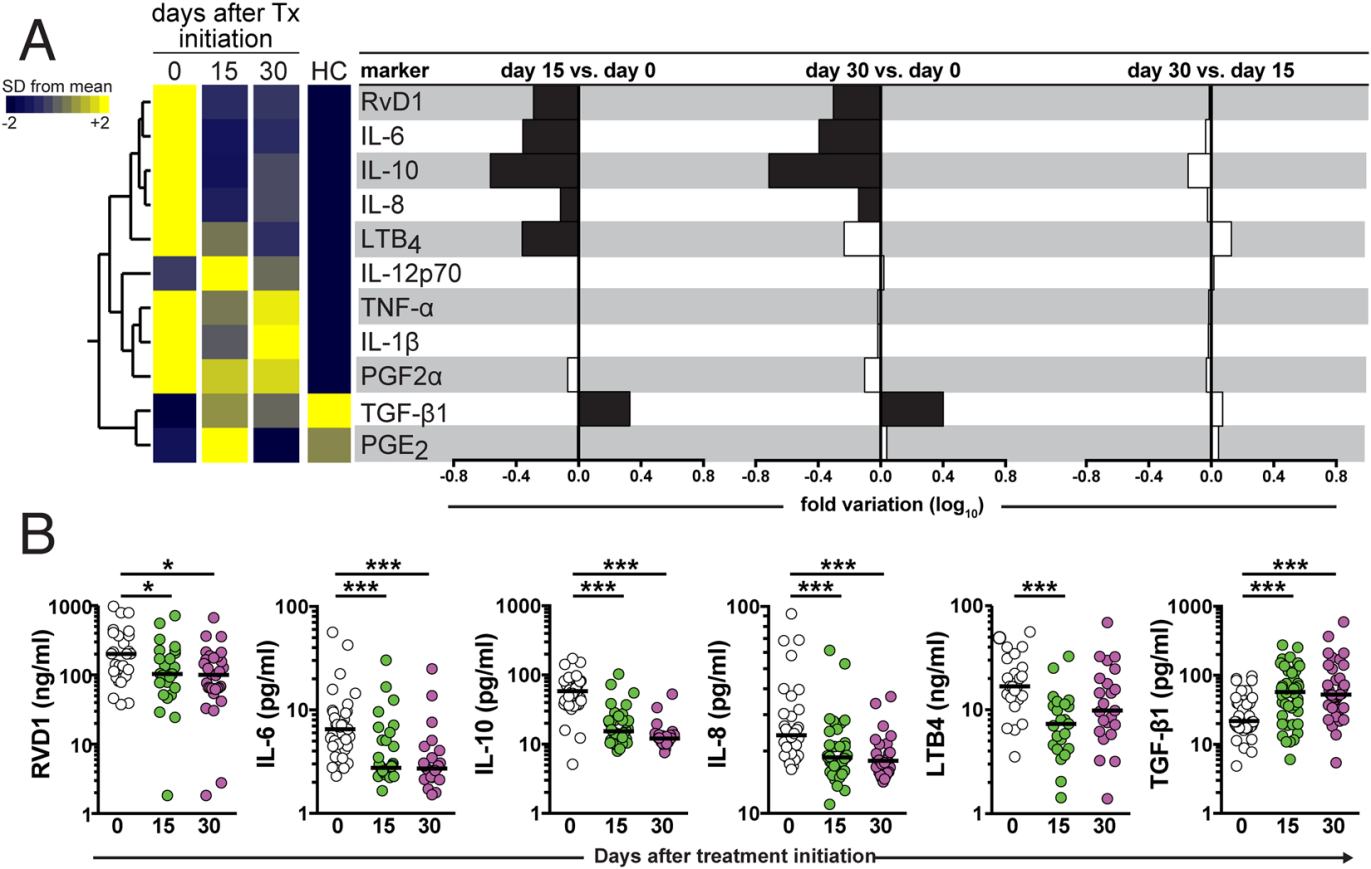
Serum concentrations of prostaglandins and cytokines in treatment-naïve patients with visceral leishmaniasis. (**A**) A hierarchical clustering analysis (Ward’s method) was employed to depict the overall expression profile of PGE_2_, PGF_2_α, LTB_4_ and RvD1, as well as the cytokines IL-1β, IL-6, IL-8, IL-10, IL-12p70, TNF-α, and TGF-β1 in serum from visceral leishmaniasis (VL) patients at different timepoints of leishmanicidal therapy and health endemic controls (HC). Fold changes were calculated and statistically significant differences are highlighted in black. (**B**) Parameters that displayed statistically significant differences between the timepoints tested by one-way ANOVA with Tukey’s post-test (after log10 transformation) are shown. Additional details of the comparisons are described in Tables 2 and 4.

**Figure 2:**
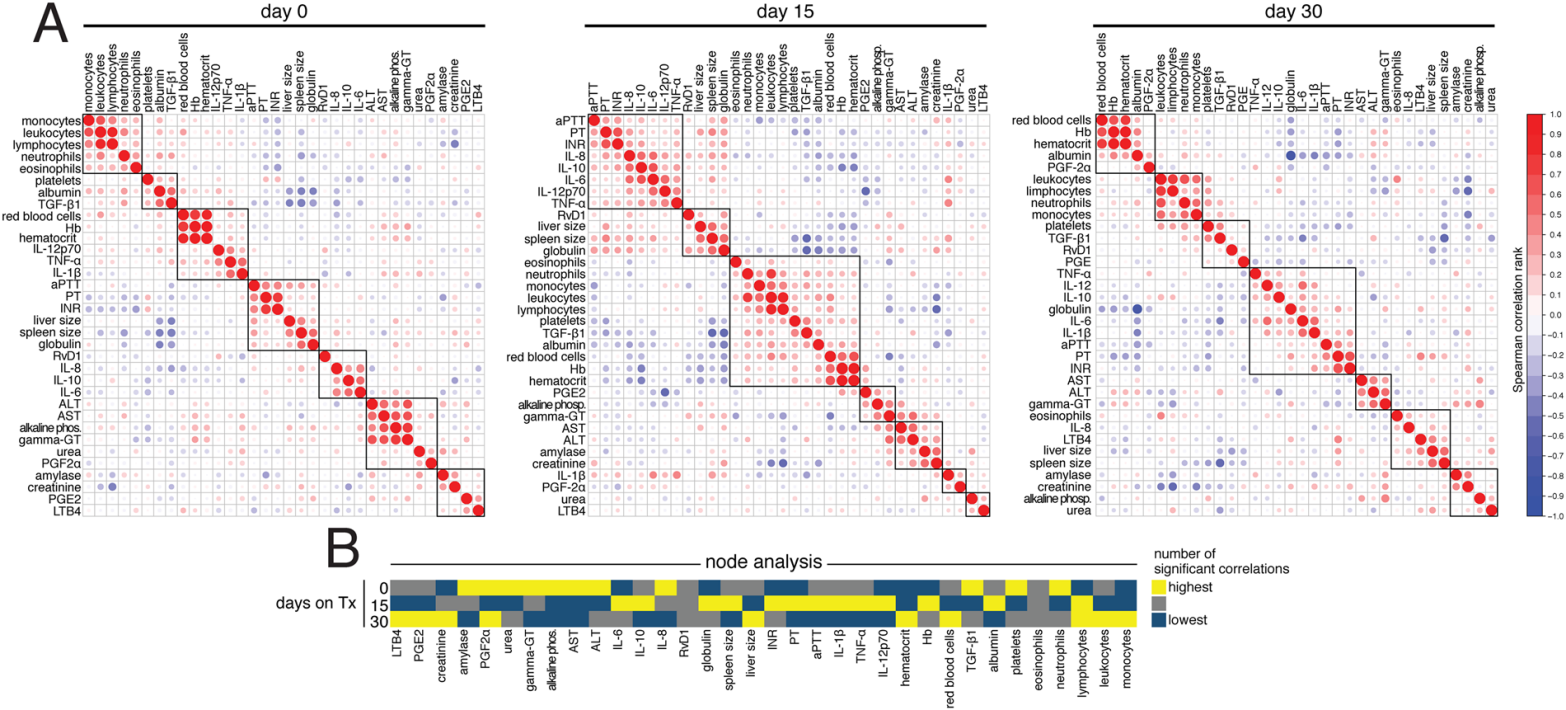
Correlation profile of inflammation and disease parameters in VL patients undergoing leishmanicidal therapy. (**A**) Heatmap shows significant correlation (p < 0.05, after adjustment for multiple measurements) of clustered Spearman matrices including serum cytokines, lipid mediators, as well as clinical, hematological, and laboratory parameters in VL patients at indicated timepoints of leishmanicidal therapy. A heatmap using the Spearman rank values was used to illustrate the matrices and only statistically significant correlations are shown. (**B**) A heatmap of the number of significant correlations involving each parameter examined is shown.

We next prospectively examined changes in serum concentrations of all the inflammatory markers in VL patients induced by initiation of leishmanicidal therapy. To do so, matched serum measurements in treatment naïve patients were compared with those performed at 15 and 30 days after initiation of anti-parasite chemotherapy (Table 3). Our results comparing healthy controls with treatment naïve VL patients revealed so far that the latter group exhibited a biosignature with a distinct expression profile of inflammatory markers in serum (Fig. 1A). A hierarchical clustering analysis of all the serum markers measured at different time points post leishmanicidal treatment initiation clearly showed that the overall expression profile in VL patients at day 30 of therapy initiation became similar to that observed in healthy controls (Fig. 1A). Amongst all the markers, TGF-β1 levels significantly increased whereas IL-10, IL-6, IL-8 and RvD1 values substantially decreased at 30 days after therapy implementation compared to pre-treatment time point (Fig. 1B and Table 4). LTB_4_ levels significantly dropped at day 15 of therapy but remained unchanged at day 30, 10 days after antimonial therapy completion (Fig. 1B). Spearman correlation matrices of the inflammatory mediators, clinical, hematological and biochemical parameters revealed that the association profiles between these factors substantially changed gradually after treatment implementation (Fig. 2A). Interestingly, at day 30 of treatment initiation, LTB_4_, PGE_2_, PGF_2_α and leukocyte counts displayed the highest number of significant correlation the matrices, suggesting a potential participation in the inflammatory environment modified by therapy (Fig. 2B). These data argue that the inflammatory profile observed during active VL is reverted upon patient recovery driven by leishmanicidal therapy.

**Table 4:** Serum levels of inflammatory markers from patients with visceral leishmaniasis before and after leishmanicidal treatment initiation.

Parameter	Units	Pre-treatment	Day 15	Day 30	P-value	Post-test result
TGF-β1	pg/mL	21.8 (17.4–38.9)	57.5 (28.1–99.4)	52.5 (33.8–123.8)	<0.0001	*, #
PGE_2_	pg/mL	0.7 (0.4–1.0)	0.7 (0.5–0.8)	0.7 (0.5–0.9)	0.157	n.s.
PGF_2_α	ng/mL	5.3 (4.0–6.5)	4.4 (3.1–6.6)	3.9 (3.0–5.6)	0.179	n.s.
LTB4	ng/mL	16.8 (12.6–29.7)	7.3 (4.2–11.3)	9.1 (6.2–17.31)	0.0321	*
RvD1	ng/mL	200 (92.3–369)	103 (58.6–217)	100 (63.4–177)	0.0171	#
TNF-α	pg/mL	21.7 (19.9–27.1)	20.8 (18.2–25.6)	21.2 (18.2–23.8)	0.362	n.s.
IL-1 β	pg/mL	17.0 (14.6–22.2)	16.0 (14.5–19.3)	15.5 (14.1–17.2)	0.702	n.s.
IL-6	pg/mL	6.5 (4.3–9.4)	2.7 (2.4–4.9)	2.7 (2.2–3.2)	<0.0001	*, #
IL-8	pg/mL	23.8 (19.2–29.8)	18.6 (16.0–21.8)	17.9 (15.8–20.6)	0.0002	*, #
IL-10	pg/mL	58.2 (37.7–78.5)	15.4 (11.5–22.2)	12.0 (10.3–13.5)	<0.0001	*, #
IL-12p70	pg/mL	8.5 (7.4–9.8)	8.7 (7.5–10.8)	8.9 (7.5–9.7)	0.508	n.s.

Values represent median and interquartile ranges. Log10 transformed data (which presented Gaussian distribution) were analyzed using one-way ANOVA with Tukey’s multiple comparisons post-test. Column with P-values represent the one-way ANOVA comparisons. P-values from post-test are represented by the following: *p < 0.05 in Pre-treatment vs. Day 15; ^#^p < 0.05 in Pre-treatment vs. Day 30; ^$^p < 0.05 in Day 15 vs. Day 30; n.s. nonsignificant.

## Discussion

The identification of pathogenesis markers in VL is warranted to identify potential disease determinants, which may serve as therapeutic targets. In the present study, performed in a highly endemic VL area in Brazil, we describe that treatment naïve VL patients exhibit a very distinct expression profile of inflammatory cytokines and lipid mediators. Previous studies have already shown a high production of proinflammatory cytokines in patients with VL^2, 3, 32^. Our results expand the panel of biomarkers likely associated with VL disease activity, incorporating lipid mediators and innate immune cytokines. Our findings reveal a distinct biosignature of active VL hallmarked by increased serum levels of PGF_2α_, LTB_4_, RvD1, TNF-α, IL-1β, IL-6 and IL-8, IL-10 and IL-12p70, while exhibiting lower concentrations of TGF-β1, compared with healthy endemic controls independent on age and gender. The inflammatory profile of cytokines, as well as the relationships between these markers and several hematological and biochemical parameters, was shown to gradually revert after leishmanicidal treatment initiation, suggesting that the expression profile was indeed induced by active disease/infection. Amongst all markers measured, TGF-β1 levels significantly increased whereas, IL-6, IL-8, IL-10 and RvD1 values substantially decreased at 30 days after therapy implementation compared to active VL disease.

The Inflammatory response during VL is characterized by increased concentrations of circulating cytokines and inflammatory mediators^2^. Our results validated previously published data showing altered levels of cytokines such as TNF-α, TGF-β1, IL-6, IL-8, IL-10 and IL-12p70 in patients with active VL, which reverted to values observed in healthy controls after anti-parasite treatment^17, 21, 27, 29, 33–35^. In addition, high levels of TNF-α, IL-1β, IL-6 and IL-8 have been independently implicated with disease severity and death associated with VL^2, 18^. Interestingly, our results demonstrated that IL-1β, a known proinflammatory cytokine involved in the activation of inflammasome in several disease models including *Leishmania* infection^36^, presented increased levels in the active VL patients, but serum concentrations did not change after therapy initiation. It is possible that the activation of IL-1β in patients with VL requires longer period after parasite clearance to be reduced. Additional mechanistic studies are necessary to better narrow the role of IL1-β-derived inflammosome activation in human VL disease.

Within the panel of markers evaluated herein, IL-10 has been systematically linked to the VL pathogenesis. Indeed, this cytokine has been considered as a key regulatory cytokine in VL due to its pleiotropic effects associated with suppression of microbicidal functions in infected macrophages^37–39^. The immunosuppressive activities of IL-10 promote parasite replication and the high levels of IL-10 observed in VL patients have been previously associated with disease progression^6, 26, 40^. Our findings showing that the heightened IL-10 levels observed in VL patients consistently decreased upon leishmanicidal treatment initiation reinforce the idea that this cytokine is indeed associated with active VL.

Although the role of cytokines in the pathogenesis of VL has been largely explored, studies reporting participation of lipid mediators in the host responses during leishmaniasis are scarce. Recently, our group has described unique expression profiles of the lipid mediator pathway in patients with tegumentary cutaneous leishmaniasis^11^. While the PGE_2_ levels were found to be increased in localized leishmaniasis compared to endemic controls, the concentrations of PGF_2_α were reported to be decreased in this same group^11^. Interestingly, the evaluation of PGE_2_ and PGF_2_α concentrations in serum from VL patients performed in the present study suggested that this disease is likely associated with an expression profile which is different from that reported in patients with localized leishmaniasis. While PGE_2_ has been described as an important biomarker distinguishing different clinical forms of tegumentary leishmaniasis such as localized and mucosal disease^11^, results presented here demonstrated that this prostanoid could not discriminate VL patients from uninfected individuals. Serum concentrations of PGE_2_ also did not significantly change upon treatment initiation. Furthermore, PGF_2_α levels were reported to be increased in active VL but may not directly reflect disease because such levels did not consistently reduce at day 30 of anti-parasite treatment. The disparities observed in eicosanoid concentrations in serum between cutaneous disease and VL may represent differences in parasite strains and/or local vs. systemic infection.

The expression profile of lipoxygenase products in leishmaniasis patients *in vivo* has been previously explored^11^. In tegumentary leishmaniasis, circulating levels of LTB_4_ and RvD1 have been described to be elevated in patients with mucosal disease, a highly inflammatory clinical form, compared to those with localized infection^11^. More recently, we have reported that RvD1 drives establishment of *Leishmania amazonensis* infection in human monocyte-derived macrophages and that its levels are increased in diffuse cutaneous leishmaniasis compared to those with localized disease^12^. These observations argue that lipoxygenase products may tightly associate with the dysfunctional disease resistance and/or tolerance in leishmaniasis. Here, we found that levels of both LTB_4_ and RvD1 were substantially increased in VL patients compared to healthy controls. Furthermore, prospective assessment of these parameters in serum revealed that concentrations of both lipid mediators were significantly reduced during leishmanicidal therapy compared to that detected at pre-treatment. LTB_4_ values decreased early following antimonial treatment (day 15), but did not further changed after 10 days of therapy completion (day 30), suggesting that this marker may read better early therapeutic response than at later timepoints. Interestingly, in dogs with VL, which were further stratified according to a clinical score^14^, we have reported that serum LTB_4_ levels gradually decreased following increased disease severity. In this setting, if LTB_4_ levels indeed reflect the degree of immune activation, dogs developing severe VL disease-associated immune suppression may exhibit reductions in its circulating values. This hypothesis needs to be tested in dogs and humans in future studies, as the present investigation did not systematically explored VL clinical severity.

We have recently described a decrease in TGF-β1 serum concentrations in localized leishmaniasis patients compared with endemic controls, while in the diffuse cutaneous leishmaniasis we observed increased values of this marker^15^. Moreover, we also described a decrease in TGF-β1 serum concentration, which was proportional to the degree of malaria disease severity^41^. Herein, we found that TGF-β1 levels were decreased in VL compared with healthy controls, and became higher after 30 days of leishmanicidal treatment, as previous described^23^. TGF-β1 has been implicated in the susceptibility to VL due to its suppressor effects on macrophages during *Leishmania* infection^30^. Nevertheless, there are few studies demonstrating the role of TGF-β1 during active VL disease^31, 42^. TGFB1 gene polymorphism (−509 C/T) was been shown in individuals with VL^19^. The relationship between the polymorphisms of TGF-β1 and other inflammatory markers described here as well as possible direct effects on parasite load remains to be addressed.

Our study reveals a distinct biosignature associated with human VL based on simultaneous assessment of several key biomarkers of inflammation in a patient cohort. Additional studies using larger patient cohorts from other endemic areas will be necessary to validate the results presented in this article.

## Electronic supplementary material

Supplemental Table 1

## References

[1] Ministério da Saúde, Brasil. *Visceral leishmaniasis: clinical recommendations for lethality reduction*. 78p (2011).

[2] Costa DL (2013). Serum cytokines associated with severity and complications of kala-azar. Pathog Glob Health.

[3] Peruhype-Magalhaes V (2005). Immune response in human visceral leishmaniasis: analysis of the correlation between innate immunity cytokine profile and disease outcome. Scand J Immunol.

[4] Peruhype-Magalhaes V (2006). Mixed inflammatory/regulatory cytokine profile marked by simultaneous raise of interferon-gamma and interleukin-10 and low frequency of tumour necrosis factor-alpha(+) monocytes are hallmarks of active human visceral Leishmaniasis due to Leishmania chagasi infection. Clin Exp Immunol.

[5] Holaday BJ (1993). Potential role for interleukin-10 in the immunosuppression associated with kala azar. J Clin Invest.

[6] Nylen S, Sacks D (2007). Interleukin-10 and the pathogenesis of human visceral leishmaniasis. Trends Immunol.

[7] Gollob KJ, Viana AG, Dutra WO (2014). Immunoregulation in human American leishmaniasis: balancing pathology and protection. Parasite Immunol.

[8] Belo VS (2014). Risk factors for adverse prognosis and death in American visceral leishmaniasis: a meta-analysis. PLoS Negl Trop Dis.

[9] Bozza PT, Bakker-Abreu I, Navarro-Xavier RA, Bandeira-Melo C (2011). Lipid body function in eicosanoid synthesis: an update. Prostaglandins Leukot Essent Fatty Acids.

[10] Dennis EA, Norris PC (2015). Eicosanoid storm in infection and inflammation. Nat Rev Immunol.

[11] Franca-Costa J (2016). Differential Expression of the Eicosanoid Pathway in Patients With Localized or Mucosal Cutaneous Leishmaniasis. J Infect Dis.

[12] Malta-Santos H (2017). Resolvin D1 drives establishment of Leishmania amazonensis infection. Sci Rep.

[13] Araujo-Santos T (2014). Role of prostaglandin F2alpha production in lipid bodies from Leishmania infantum chagasi: insights on virulence. J Infect Dis.

[14] Solca MS (2016). Circulating Biomarkers of Immune Activation, Oxidative Stress and Inflammation Characterize Severe Canine Visceral Leishmaniasis. Sci Rep.

[15] Franca-Costa J (2015). Arginase I, polyamine, and prostaglandin E2 pathways suppress the inflammatory response and contribute to diffuse cutaneous leishmaniasis. J Infect Dis.

[16] Ansari NA, Saluja S, Salotra P (2006). Elevated levels of interferon-gamma, interleukin-10, and interleukin-6 during active disease in Indian kala azar. Clin Immunol.

[17] van der Poll T, Zijlstra EE, Mevissen M (1995). Interleukin 6 during active visceral leishmaniasis and after treatment. Clin Immunol Immunopathol.

[18] Dos Santos PL (2016). The Severity of Visceral Leishmaniasis Correlates with Elevated Levels of Serum IL-6, IL-27 and sCD14. PLoS Negl Trop Dis.

[19] Frade AF (2011). TGFB1 and IL8 gene polymorphisms and susceptibility to visceral leishmaniasis. Infect Genet Evol.

[20] Karp CL (1993). *In vivo* cytokine profiles in patients with kala-azar. Marked elevation of both interleukin-10 and interferon-gamma. J Clin Invest.

[21] de Medeiros IM, Castelo A, Salomao R (1998). Presence of circulating levels of interferon-gamma, interleukin-10 and tumor necrosis factor-alpha in patients with visceral leishmaniasis. Rev Inst Med Trop Sao Paulo.

[22] Bacellar O, D’Oliveira A, Jeronimo S, Carvalho EM (2000). IL-10 and IL-12 are the main regulatory cytokines in visceral leishmaniasis. Cytokine.

[23] Caldas A (2005). Balance of IL-10 and interferon-gamma plasma levels in human visceral leishmaniasis: implications in the pathogenesis. BMC Infect Dis.

[24] Saha S (2007). IL-10- and TGF-beta-mediated susceptibility in kala-azar and post-kala-azar dermal leishmaniasis: the significance of amphotericin B in the control of Leishmania donovani infection in India. J Immunol.

[25] Luz NF (2012). Heme oxygenase-1 promotes the persistence of Leishmania chagasi infection. J Immunol.

[26] Gautam S (2011). IL-10 neutralization promotes parasite clearance in splenic aspirate cells from patients with visceral leishmaniasis. J Infect Dis.

[27] Barral-Netto M (1991). Tumor necrosis factor (cachectin) in human visceral leishmaniasis. J Infect Dis.

[28] Medeiros IM, Reed S, Castelo A, Salomao R (2000). Circulating levels of sTNFR and discrepancy between cytotoxicity and immunoreactivity of TNF-alpha in patients with visceral leishmaniasis. Clin Microbiol Infect.

[29] Salomao R, Castelo Filho A, de Medeiros IM, Sicolo MA (1996). Plasma levels of tumor necrosis factor-alpha in patients with visceral leishmaniasis (Kala-azar). Association with activity of the disease and clinical remission following antimonial therapy. Rev Inst Med Trop Sao Paulo.

[30] Barral-Netto M (1992). Transforming growth factor-beta in leishmanial infection: a parasite escape mechanism. Science.

[31] Rodrigues V, Santana da Silva J, Campos-Neto A (1998). Transforming growth factor beta and immunosuppression in experimental visceral leishmaniasis. Infect Immun.

[32] Costa AS (2012). Cytokines and visceral leishmaniasis: a comparison of plasma cytokine profiles between the clinical forms of visceral leishmaniasis. Mem Inst Oswaldo Cruz.

[33] Bacellar O, Barral-Netto M, Badaro R, Carvalho EM (1991). Gamma interferon production by lymphocytes from children infected with L. chagasi. Braz J Med Biol Res.

[34] Hailu A, van der Poll T, Berhe N, Kager PA (2004). Elevated plasma levels of interferon (IFN)-gamma, IFN-gamma inducing cytokines, and IFN-gamma inducible CXC chemokines in visceral leishmaniasis. Am J Trop Med Hyg.

[35] Kurkjian KM (2006). Multiplex analysis of circulating cytokines in the sera of patients with different clinical forms of visceral leishmaniasis. Cytometry A.

[36] Lima-Junior DS (2013). Inflammasome-derived IL-1beta production induces nitric oxide-mediated resistance to Leishmania. Nat Med.

[37] Gazzinelli RT, Oswald IP, James SL, Sher A (1992). IL-10 inhibits parasite killing and nitrogen oxide production by IFN-gamma-activated macrophages. J Immunol.

[38] Bogdan C, Vodovotz Y, Nathan C (1991). Macrophage deactivation by interleukin 10. J Exp Med.

[39] Oswald IP, Wynn TA, Sher A, James SL (1992). Interleukin 10 inhibits macrophage microbicidal activity by blocking the endogenous production of tumor necrosis factor alpha required as a costimulatory factor for interferon gamma-induced activation. Proc Natl Acad Sci U S A.

[40] Kumar R, Nylen S (2012). Immunobiology of visceral leishmaniasis. Front Immunol.

[41] Andrade BB (2010). Heme impairs prostaglandin E2 and TGF-beta production by human mononuclear cells via Cu/Zn superoxide dismutase: insight into the pathogenesis of severe malaria. J Immunol.

[42] Gomes NA, Gattass CR, Barreto-De-Souza V, Wilson ME, DosReis GA (2000). TGF-beta mediates CTLA-4 suppression of cellular immunity in murine kalaazar. J Immunol.

