# Supplementary material for: Anti-parasite therapy drives changes in human visceral leishmaniasis-associated inflammatory balance
Source: Sci Rep. 2017 Jun 28;7:4334. doi: 10.1038/s41598-017-04595-8 (PMC5489532; doi:10.1038/s41598-017-04595-8)
Supplement: Supplementary file 1 — Supplemental Table 1 [file 41598_2017_4595_MOESM1_ESM.doc]

**Anti-parasite therapy drives changes in human visceral leishmaniasis-associated inflammatory balance**

Théo Araújo-Santos, Bruno B. Andrade, Leonardo Gil-Santana, Nívea F. Luz, Priscila L. dos Santos, Fabrícia A. de Oliveira; Meirielly Lima Almeida, Roseane Nunes de Santana Campos, Patrícia T. Bozza; Roque P. Almeida &Valéria M. Borges

**Supplemental Table 1. Distribution of biomarker values by gender.**

Data were compared using the Mann-Whitney *U* test.

| Parameter | Unit | Healthy Control | | | Visceral Leishmaniasis | | |
| --- | --- | --- | --- | --- | --- | --- | --- |
|  |  | Female | Male | P-value | Female | Male | P-value |
| TGF-β1 | pg/mL | 56.5 (43.8-67.1) | 48.2 (45.2-58.5) | 0.3637 | 21.1 (17.4-38.3) | 20.5 (14.0-38.8) | 0.5629 |
| PGE_2_ | pg/mL | 0.7 (0.5-0.9) | 0.54 (0.3-0.9) | 0.2665 | 0.6 (0.3-1.0) | 0.7 (0.5-1.0) | 0.7666 |
| PGF_2_α | ng/mL | 0.6 (0.4-0.7) | 0.3 (0.2-0.5) | 0.0513 | 4.7 (3.0-7.6) | 5.6 (4.5-7.1) | 0.3152 |
| LTB_4_ | ng/mL | 5.3 (4.1-10.4) | 4.0 (2.5-4.3) | 0.0761 | 13.1 (9.0-32.8) | 20.1 (15.0-32.0) | 0.2432 |
| RvD1 | ng/mL | 89.5 (34.6-124.4) | 64.0 (47.0-103.1) | 0.6727 | 171.0 (93.6-396.0) | 291.1 (89.0-434.5) | 0.4227 |
| TNF-α | pg/mL | 0.97 (0.7-1.8) | 1.8 (0.9-5.0) | 0.1023 | 19.9 (18.0-24.5) | 21.7 (20.5-27.9) | 0.0990 |
| IL-1β | pg/mL | 0.9 (0.4-1.3) | 1.1 (0.8-1.7) | 0.2468 | 16.8 (14.5-21.1) | 16.3 (15.0-22.2) | 0.8142 |
| IL-6 | pg/mL | 1.3 (0.9-2.3) | 1.9 (1.0-5.7) | 0.4250 | 6.9 (3.5-11.4) | 5.3 (4.1-9.3) | 0.6017 |
| IL-8 | pg/mL | 8.4 (6.4-12.9) | 10.2 (6.1-73.4) | 0.4020 | 23.7 (18.9-37.4) | 25.4 (19.5-36.9) | 0.7439 |
| IL-10 | pg/mL | 2.0 (1.6-3.0) | 2.5 (2.0-26.4) | 0.1276 | 59.6 (37.1-94.9) | 58.3 (38.1-82.0) | 0.7788 |
| IL-12p70 | pg/mL | 1.8 (1.5-2.8) | 2.6 (2.2-3.4) | 0.0677 | 8.7 (7.0-9.7) | 8.5 (7.9-10.2) | 0.4585 |
